# Chitosan Encapsulation of Ferrate^VI^ for Controlled Release to Water:Mechanistic Insights and Degradation of Organic Contaminant

**DOI:** 10.1038/s41598-019-54798-4

**Published:** 2019-12-04

**Authors:** Bo-Yen Chen, Hsuen-Wen Kuo, Virender K. Sharma, Walter Den

**Affiliations:** 1 Department of Environmental Science and Engineering, Tunghai University, Taichung, Taiwan ROC; 2 Department of Environmental and Occupational Health, School of Public Health, Texas A&M University, College Station, Texas USA; 3 Institute for Water Resources Science and Technology, Department of Science and Mathematics, Texas A&M University-San Antonio, San Antonio, Texas USA

**Keywords:** Pollution remediation, Chemical engineering

## Abstract

Tetraoxy-anion of iron in +6 oxidation state (Fe^VI^O_4_^2−^, Fe^VI^), commonly called ferrate, has shown tremendous potential as a green oxidative agent for decontaminating water and air. Encapsulation of solid potassium salt of ferrate (K_2_FeO_4_) circumvents the inherent drawbacks of the instability of ferrate under humid conditions. In the encapsulated strategy, controlled release without exposing the solid ferrate to the humid environment avoids self-decomposition of the oxidant by water in the air, and the ferrate is mostly used to decontaminate water efficiently. This study demonstrated the formulation of oxidative microcapsules with natural materials present in chitosan, whose release rate of the core material can be controlled by the type of intermediate hydrocarbon layer and the pH-dependent swelling of chitosan shell. The pH played a pivotal role in swelling chitosan shell and releasing the core oxidant. In a strong acidic solution, chitosan tended to swell quickly and release Fe^VI^ at a faster rate than under neutral conditions. Additionally, among the several long-chain hydrocarbon compounds, oleic acid exhibited the strongest “locking” effect when applied as the intermediate layer, giving rise to the slow release of Fe^VI^. Coconut oil and mineral oil, in comparison, allowed Fe^VI^ to penetrate the layer within shorter lengths of time and showed comparable degrees of degradation of target contaminant, methylene orange, under ambient temperature and near-neutral conditions. These findings have practical ramifications for remediating environmental and industrial processes.

## Introduction

The natural abundance of iron renders the iron-based technologies a highly desirable approach for decontaminating the environment because they do not introduce synthetic material or harmful by-products foreign to natural environment^1–5^. In particular, simple tetra-oxy anion of high-valent iron (Fe^VI^O_4_^2−^, Fe^VI^), usually called ferrate, has exhibited a strong oxidative power and effectiveness for treating a wide range of environmental contaminants^6–10^. Fe^VI^ possesses a greater redox potential in acidic solutions than other common oxidizing agents (hydrogen peroxide, hypochlorite, perchlorate) for disinfection^11,12^ and depolluting water^13^. As a microbial disinfectant, Fe^VI^ had demonstrated 3-log kill rates of total coliforms and chlorine-resistant bacteria from the genera Bacillus and Mycobacterium^14^, and required less time to inactivate *Escherichia coli* at lower dosages than hypochlorite^15^. The acidity of Fe^VI^ species plays a prominent role in its effectiveness to inactivate *E. coli*, as the rate constant of *E. coli* inactivation by Fe^VI^ nearly doubles when pH reduces from 8.2 to 5.6^16^. Researchers have also demonstrated the multiple roles of Fe^VI^ as a coagulant and oxidant in removing metals, nutrients, radionuclides, humic acids^17–21^ and emerging micropollutants (e.g., estrogenic hormone^22,23^, antibiotics^24,25^, and other pharmaceutical ingredients^26^) in water. Recently, studies have shown that the activated ferrate shortened the reaction time for transforming emerging pollutants that can only be partially be oxidized by Fe^VI^ (or un-activated ferrate)^27–31^.

Despite being a powerful oxidant, the practical applications of ferrate are very limited. The studies demonstrating ferrate as an effective chemical to decontaminate have used a solid K_2_FeO_4_, which is unstable under humid and lit conditions. In large-scale and long-term use in applications, the solid K_2_FeO_4_ must be stored under special conditions with no light exposure and no water in the air (or anoxic conditions)^32,33^. These conditions restrict applications of ferrate to treat environmental pollutants under ambient conditions. In the present paper, we have made a major stride to overcome issues associated with the instability of ferrate by using the encapsulation strategy that could store a solid salt of K_2_FeO_4_ without exposure to environmental stimuli (e.g., light, moisture, oxygen). Additionally, encapsulation could release the ferrate in a controlled manner to the intended applications. Furthermore, encapsulation was carried out by developing an architecture that encapsulates ferrate with natural-derived material in chitosan.

Studies on encapsulation of ferrate in the literature are scant^34,35^. Only two studies have been reported on encapsulating ferrate, which used paraffin wax as a shell material. Paraffin is a mixture of saturated hydrocarbons and does not fall in the category of green material. Therefore, we searched for natural material and have selected chitosan. We hypothesised that the molecular structure of chitosan could be altered to control the release rate. Additionally, chitosan is chemically stable (e.g., high thermal stability up to 280 °C) and owns a distinct advantage over other encapsulating agents: the possibility to establish covalent or ionic bonds with the crosslinking agents, hence building a network structure to retain the active substance. Consequently, these chemical bonds carry advantages regarding controlled release^36,37^. Ionically crosslinked microparticles form non-permanent and reversible networks, allowing chitosan microparticles to exhibit a higher swelling sensitivity to pH changes as compared to the covalently crosslinked counterpart. This property extends its potential application since dissolution can occur in extremely acidic or basic pH conditions^38^. Crosslinking agents like tripolyphosphate, citrate, sulfate, and phosphate, among others, are used^39,40^. To avoid unintended reaction between the active reagent (Fe^VI^) and the binder (chitosan), an intermediate layer to stabilise Fe^VI^ may be necessary.

The specific objectives of the current paper are: (i) to evaluate the effectiveness of various types of oleochemical (oleic acid and coconut oil) and petrochemical (mineral oil) agents as the buffering medium; (ii) to examine the pH effect on the release behavior of Fe^VI^ from microencapsulation; and (iii) to demonstrate the functionality of the encapsulated Fe^VI^ to remove contaminants by investigating degradation of methyl orange as the model contaminant.

## Results and Discussion

### Encapsulation parameters and material characterization

The parameters investigated in the encapsulation studies included the concentration of chitosan in 1% acetic acid solution, the type (i.e., NaOH and KOH) and concentration of the hardening solution, and the kind and concentration of additives used as the buffering agent. The resultant capsules were visually examined to assess the optimum combination of chemicals to be used. In brief, chitosan concentration less than 1% did not yield sufficient wall formation to encapsulate Fe^VI^ completely, resulting in a rapid decomposition of Fe^VI^ to Fe^III^. Chitosan concentration at 1.0% and 1.5% provided the improved formation of the wall. The concentration at 2.0%, however, yielded the incomplete solid wall after hardening. Furthermore, NaOH generally performed better as a hardening agent for chitosan than KOH, with 10% and 15% as an appropriate range of concentration. Among the buffering agent, the fatty acid organic molecules in oleic acid were favorable to form a protective layer around solid particles of ferrate. Comparatively, long-chain alkanal (aldehyde) was not able to have a protective layer.

The SEM images, shown in Fig. S1, of the encapsulated K_2_FeO_4_ samples revealed that the thickness of the wall material fabricated with 0.5% chitosan solution was about 0.31 mm, and those with 1.0% and 1.5% chitosan solution was about 0.375 mm. The SEM image that the encapsulated ferrate cladding material was exposed to change the pH of the hardening solution, resulting in the coacervation between the chitosan and the alkaline solution to produce a crystalline material. The energy dispersive spectra further verified the presence of a higher level of carbon (57.2% wt mainly due to chitosan, oleic acid, and carbon tape artifact), iron (16.5% wt) and potassium (11.1% wt). The remaining elements are sodium (9.1% wt due to the use of hardening solution by sodium hydroxide) and oxygen (6.1% wt).

Applying different types of buffering agents appeared to result in slightly different pellet sizes. With oleic acid, the capsule size ranged between 0.4 and 0.5 mm. The SEM image clearly shows the formation of chitosan shell wall with a typical thickness of around 0.1–0.2 mm. Addition of a surfactant to enhance mixing between Fe^VI^ and oleic acid resulted in smaller capsules (0.2–0.3 mm), without a clear distinction between the shell wall and the inner content.

With coconut oil being the buffering agent, the size of the capsules yielded was similar to those observed in Fe^VI^/OA/chitosan using Tween 80. A two-step process was performed to synthesise the capsules, namely a first step to mix Fe^VI^ with liquid coconut oil before hardening by temperature reduction, and a second step to further coated with chitosan. While the image shows the transparent oily layer covering solid K_2_FeO_4_, the freeze-dried capsule did not yield a clear presence of shell wall of chitosan. The result indicates that the dip-coating method applied in the second step may need to be modified to ensure the attachment of chitosan to the droplets from a shell wall.

The XRD pattern (Fig. S2) of the encapsulated ferrate contains the same characteristic peaks as that of K_2_FeO_4_ with an orthorhombic unit cell possessing the spaces group D2h, but with a lower intensity. The XRD characteristic peaks of as-obtained K_2_FeO_4_ at 2θ = 20.9° and 30° correspond to the (111) and (031) phases of the crystalline. The XRD patterns of standard chitosan procured from the manufacturer show similar peaks as the peaks of 2θ between 18° and 20° were related to the crystal in chitosan structure^41^.

The transmittance FT-IR spectra (Fig. S3) of the as-received K_2_FeO_4_ and the encapsulated ferrate samples include a primary peak at 825 cm^−1^, which is characteristic of FeO_4_^2−^ attributed to the stretching vibrations of the FeO bond. The spectra of chitosan show a broad absorption band in the region of 3,450 cm^−1^ that corresponds to the OH stretching vibrations of water and hydroxyls and the NH stretching vibrations of the free amino group. The bands observed at 2,924 and 2,852 cm^−1^ correspond to the asymmetric stretching of CH_3_ and CH_2_ in the chitosan. The intensive peak at 1,629 cm^−1^ corresponds to the bending vibration of NH_2_ characteristic of chitosan polysaccharide, indicating the occurrence of deacetylation^41^.

### Formation and stability of capsules with different buffering agents

The reaction between Fe^VI^ and chitosan was verified by titrating 0.5% (w/v in acetic acid) to a solution containing up to 400 mg/l of K_2_FeO_4_. The linear correlation (Fig. S4) between the amount of chitosan needed to reduce Fe^VI^ to Fe^III^ indicates a stoichiometric reaction and justifies the necessity to add a buffer layer between the wall material and the oxidant. Each unit of chitosan molecule contains glucosamine (Glc-NH_2_) linked by β(1,4) bond (hence forming 1,4-β-D-glucoside bond). To the best of our knowledge, there has not been any literature available concerning the chitosan oxidation by ferrate. Depolymerization of chitosan molecules via the breakage of the β(1,4) bonds, however, is a strong possibility given the previous works reported involving the use of other oxidants such as ozone and hydrogen peroxide^42,43^. When exposing the as-prepared encapsulated ferrate pellets in ambient air at a controlled temperature of 25 °C, the amount of potassium ferrate in the OA-buffered chitosan pellets remained consistent (Fig. S5) over a period of 20 days, suggesting that the encapsulation could effectively preserve ferrate in the ambient air environment for at least 20 days. Some degree of variability existed because the actual amount of potassium ferrate contained in each pellet varied. We estimated an amount of K_2_FeO_4_ between 20 mg to 40 mg was covered in the chitosan/buffer material.

**Figure 1:**
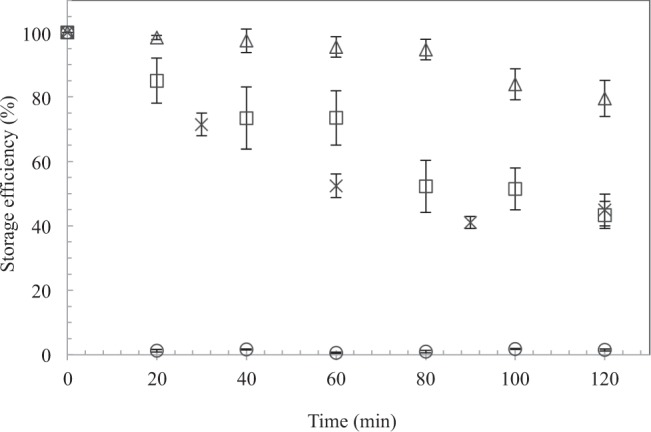
The storage capacity of oleic acid (◇), coconut oil (liquid, **□**; droplet, **×**), and mineral oil (○) as a function of time.

As for the stability of Fe^VI^ in the three types of buffering medium, Fig. 1 shows the amount of Fe^VI^ remained in a buffering agent (i.e., storage capacity) as a function of time. The results indicate that there was essentially no release of Fe^VI^ observed with OA being the buffer, even after 120 min of exposure time. This result is consistent with the open-air exposure study reported earlier where the OA-buffered chitosan pellets remained stable after 20 days. OA is the most abundant form of monounsaturated fatty acid derived from plants and animals, and the β-oxidation (β position of a two-carbon bond) pathway of OA enzymatically has been of biochemical important in food and medical sciences and chemocatalytically in the chemical process using oil and fats as the feedstock for renewable energy^44^. The result also implies that OA appears to be resistant to ferrate oxidation and hence can serve as a good storage medium for Fe^VI^. The addition of the surfactant in an attempt to enhance the dissolution of OA in the chitosan solution did not improve the release of Fe^VI^ from OA. In comparison, Fe^VI^ gradually releases when entrapped by MO, as about 90% K_2_FeO_4_ concentration remained after placing the mixture in water, and about 75% remained after 120 min. The addition of the surfactant to the MO mixture accelerated the release of K_2_FeO_4_. For CO as the buffer medium, about 40% of the entrapped Fe^VI^ was lost after 60 min and 60% lost after 120 min. Both liquid and solid form (droplet) performed similarly. CO differs from OA medium in that CO contains mostly saturated fatty acids as opposed to the large fraction of monounsaturated fatty acids in OA. Based on the chemical release study, buffering Fe^VI^ with media containing saturated fatty acids are prone to short-term release as compared to those with unsaturated fatty acids. With their variation of molecular structure, these hydrocarbon media may display the potential functionality to regulate the release rate of K_2_FeO_4_.

It is noteworthy to mention that MO is a lighter version (i.e., lesser molecular weight) of paraffin (solid) and petroleum jelly (semi-solid), all of which are a by-product of refining crude oil to make gasoline and other petroleum products. The previous studies involving the use of paraffin wax as the wall material encapsulating K_2_FeO_4_ revealed the gradual release of Fe^VI^ over time, losing about 15% of entrapped after 24 h^35^. We did not perform a release experiment for longer than two hours. Our results using MO to trap K_2_FeO_4_ largely agreed with theirs but exhibited a faster release considering the lesser density of MO as compared to paraffin wax.

**Figure 2:**
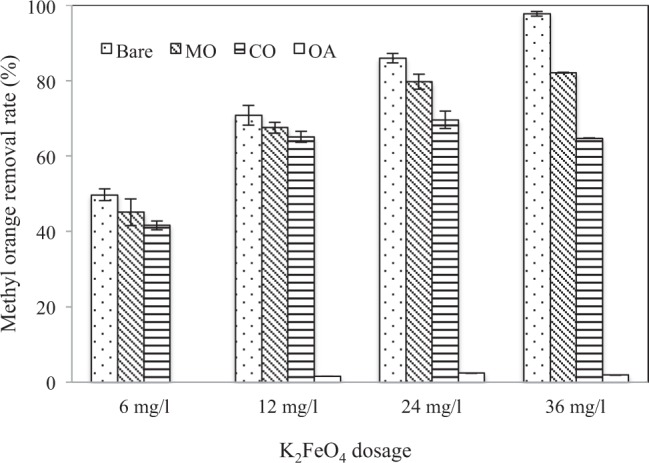
The removal of methyl orange after 20 min of reaction with the various dosage of K_2_FeO_4_ entrapped by MO, CO, and OA without chitosan encapsulation (initial pH at 6.5).

Figure 2 shows the degree of removal of methyl orange (initial concentration of 5 mg/l) using different dosages of K_2_FeO_4_ (from 0 to 36 mg/l) entrapped in OA, CO, and MO. Though the oxidative mechanism was not studied in this work, it was hypothesised that the decoloration of methyl orange solution during Fe^VI^ oxidation was a result of the bond cleavage of the chromophore groups (–N=N–)^45,46^ which are functional to the color of the appearance of methyl orange molecules. The intermediate products during the decomposition reported in the literature^47^ include N,N-dimethylbenzylamine (C_8_H_11_N), N,N-dimethyl-p-phenylenediamine (C_8_H_12_N_2_) and sulfanilic acid (C_6_H_6_NSO_3_). These intermediate compounds may be further oxidised into small molecule organics or mineralised. The pseudo-first-order reaction rate constants are 0.044/min, 0.074/min, 0.110/min, and 0.165/min for K_2_FeO_4_ dosage of 6 mg/l, 12 mg/l, 24 mg/l, and 36 mg/l (Fig. S8), respectively The linear increase in the value of the kinetic constant with the K_2_FeO_4_ dosage suggests that the experiments were predominantly reaction-limited (i.e., the rate of reaction markedly increases with higher Fe^VI^ content).

The entrapped K_2_FeO_4_ with MO and CO also yielded high degrees of removal of methyl orange, as seen in Fig. 2 (the time-dependent removal profiles of methyl orange using the various K_2_FeO_4_ dosages are shown in Fig. S9). For all challenged methyl orange concentrations, K_2_FeO_4_/MO system consistently produced higher removal rates than K_2_FeO_4_/CO, with the gap widening as the initial concentration of methyl orange increased. These results suggest two important properties: (i) The extent of methyl orange removal reflects the Fe^VI^ storage capacity displayed in Fig. 1, which shows an enhanced retainment with MO as compared to CO. (ii) Noting the rates of the methyl orange removal only marginally less than those obtained with direct Fe^VI^ oxidation (i.e., bare K_2_FeO_4_), both MO and CO were capable of retaining a large fraction of the oxidative power of Fe^VI^, exhibiting the suitability to act as the buffering medium of choice when synthesizing Fe^VI^ capsules. Contrarily, when entrapped with OA, there was a very limited degree of oxidation of methyl orange. This result was also consistent with that in the Fe^VI^ release study mentioned earlier, where very little Fe^VI^ was detected after 120 min. Given the lack of chemical reactions between Fe^VI^ and the other two types of nonpolar, long-chain hydrocarbon media, it could be inferred that oleic acid was unlikely to react with Fe^VI^ but prevented it from releasing within the length of time tested.

### Removal of methyl orange by chitosan-encapsulated Fe^VI^

**Figure 3:**
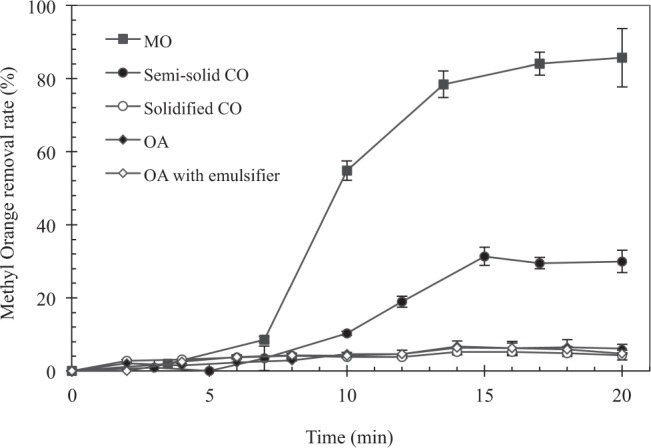
The removal of methyl orange as a function of time by Fe^VI^ encapsulated pellets using the various types of intermediate buffering media. The initial pH value was 6.5 and the initial methyl orange concentration was 5.0 mg/l.

**Figure 4:**
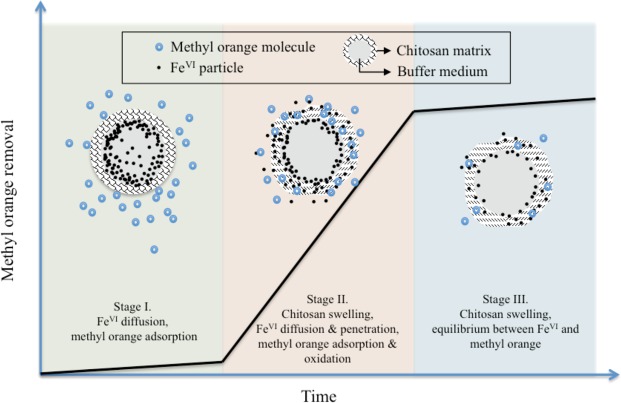
Schematic presentation of the proposal mechanism of methyl orange oxidation by encapsulated pellets.

As the chitosan-encapsulated pellets were exposed in methyl orange solutions, Fe^VI^ diffuses through the buffering medium, and the chitosan shell is subject to oxidation of methyl orange. Fig. 3 shows a typical set of methyl orange removal rate as a function of time for the various buffering media at pH 6.5. Pellets with OA as the buffering medium exhibited less than 6% of methyl orange degradation, regardless of whether OA was emulsified. Pellets prepared with MO as the buffer medium showed significant removal of methyl orange, achieving about 80% removal efficiency after 20 min. Those prepared with CO also resulted in > 30% removal. These results were consistent with the Fe^VI^ release study demonstrating that the rate of release of Fe^VI^ was regulated by its buffer medium. Furthermore, it is noted that all time-dependent profiles display a near S-shape pattern, characterised by an inert starting period (i.e., initial five minutes) in which a limited extent of methyl orange removal can be observed. This was followed by a rapid rate of increase of the methyl orange removal in the case of the chitosan/MO/K_2_FeO_4_ and chitosan/CO/K_2_FeO_4_ pellets, before reaching a state of equilibrium. This “three-stage” pattern of the removal profiles was distinct from those observed from the time-dependent Fe^VI^ release profiles (Fig. S9) which did not exhibit the initial lag period stemming from the presence of chitosan. We therefore propose a working hypothesis on the mechanism of Fe^VI^ release and oxidation that is schematically presented in Fig. 4 and delineated as follows: Initially, as the encapsulated pellets are exposed to a solution containing methyl orange, the methyl orange adsorb onto the surface of the chitosan shell, which gradually begins swelling up (Stage I). Fe^VI^ particles then diffuse out of the buffer layer by the concentration gradient and migrate along the swelled surface of chitosan to attack the adsorbed methyl orange molecules. Some particles may find their way into the solution and oxidise aqueous phase molecules (Stage II). As the available Fe^VI^ depletes, reaction with methyl orange molecules slows down, and the aqueous phase concentration eventually reaches a new state of adsorption equilibrium with chitosan shell.

### Effect of pH on methyl orange removal by Fe^VI^ pellets

A multitude of factors are involved with the removal of methyl orange under different pH values, including: (i) chitosan adsorptivity of methyl orange; (ii) swelling effect of chitosan; and (iii) Fe^VI^ reaction with methyl orange once released from the capsules. Chitosan has been known as an excellent natural sorbent for a wide range of environmental contaminants and achieved enhanced adsorption capacities under the acidic environment^48,49^. Its role as the outer shell of the encapsulation can also be partially responsible for the reduction of methyl orange in solutions. To verify, we conducted a separate set of study to measure the adsorptivity of methyl orange on chitosan, assuming the protonated amino groups on chitosan molecules can play an important role in covalent link with the sulfate branch on a methyl orange molecule (Fig. S6). Figure S7 shows a set of typical adsorption equilibrium profiles of methyl orange at two different initial concentrations (5 mg/l and 30 mg/l) on chitosan. The adsorption parameters obtained for the Langmuir isotherm model and the Freundlich isotherm model under different pH values are summarised in Table 1. The adsorption equilibrium data fit well with either model through regression analyses using the linearised form of the model equations. With K_L_ representing the adsorption capacity for the Langmuir isotherm model, an increase in the pH value resulted in reduced adsorption capacity. The decreasing K_F_ value also expresses similar results in the Freundlich isotherm. The theoretical maximum adsorption capacity (K_L_) was pH-dependent, with a value of 20.5 mg/g at pH 5.0, 9.61 mg/g at pH 7.2, and 8.81 mg/g at pH 10.0. Deprotonation of the amino ions on chitosan molecules in more basic conditions may reduce the availability of the ionic sites that are favorable for adsorption of methyl orange molecules.

**Table 1:** Adsorption isotherm constants calculated from isotherm curves

	Langmuir isotherm			Freundlich isotherm		
pH	K_L_ (mg/g)	b	R^2^	1/n	K_F_ ((mg/g)/(l/mg)^n^)	R^2^
5.0	20.5	0.0005	0.992	0.9337	0.0495	0.981
6.5	9.61	0.0037	0.988	0.9105	0.0428	0.960
10	8.81	0.0051	0.995	1.0168	0.0096	0.989

Langmuir isotherm equation: ${q}_{e}=\frac{{K}_{L}b{C}_{e}}{1+b{C}_{e}};$ q_e_ is the surface-bound mass (mg/g); C_e_ is the aqueous-phase adsorbate concentration (mg/l); K_L_ is the adsorption capacity (mg/g); b is a dimensionless constant.

Linearised form: $$\frac{1}{{q}_{e}}=\frac{1}{{C}_{e}}(\frac{1}{{K}_{L}b})+\frac{1}{{K}_{L}}$$.

Freundlich isotherm equation: ${q}_{e}={K}_{F}{C}_{e}^{1/n};$ K_F_ and n are both empirical constants.

Linearised form: $$\log \,{q}_{e}=\,\log \,{K}_{F}+\frac{1}{n}\,\log \,{C}_{e}$$

Chitosan with a p*K*a of 6.3 is polycationic when dissolved in acid and presents –NH_3_^+^ sites. At a pH value below 5, almost 90% of active sites were protonated, while at pH 4, more than 99% were protonated^50^. The increase in the positive charges on the chitosan molecule, in turn, causes a strong electrostatic repulsive force with adjacent molecules, thereby stretching a chitosan molecule. At a higher pH value, the hydroxyl ions are linked to the amino groups by deprotonation and consume the ionic sites on chitosan. Therefore, exposing chitosan in acidic conditions typically induces a greater extent of the swelling effect, which plays an important role in controlling the rate of release of the active ingredient entrapped in chitosan outer wall.

We have already demonstrated that the protonation of amino functional groups under acidic conditions would favor the adsorption of methyl orange via the ionic bonding with the sulfate group in a methyl orange molecule (point i). Similarly, protonation of amino groups also generates a repulsive electrical force that stretches the bonds in a chitosan molecule and causes a greater swelling effect (point ii).

For oxidation of methyl orange (point iii), the rate constants of reactions of these compounds with ferrate usually decrease with an increase in pH in alkaline media. Sharma reported the rate constants for the reactions of HFeO_4_^−^ and HFeO_4_^2−^ with the substance were correlated with 1-e^−^ and 2-e^−^ reduction potentials in order to understand the mechanisms of the reactions. Fe^V^ generally oxidises compounds by a 2e^−^ transfer step. The reaction of Fe^VI^ with compounds may be characterised most commonly by (i) a 1-e^−^ transfer step from Fe^VI^ to Fe^V^, followed by a 2-e^−^ transfer to Fe^III^ as the reduced product (Fe^VI^ → Fe^V^ → Fe^III^), and (ii) 2-e^−^ transfer steps (Fe^VI^ → Fe^IV^ → Fe^II^). Oxygen atom transfer to the compounds may occur through the involvement of either Fe^VI^ or Fe^V^ in the oxidations carried out by ferrate^20,51^. In summary, exposing the chitosan encapsulated pellets under an acidic condition strongly favors the removal of methyl orange because chitosan adsorbs better and swells better, while Fe^VI^ oxidises methyl orange faster.

**Figure 5:**
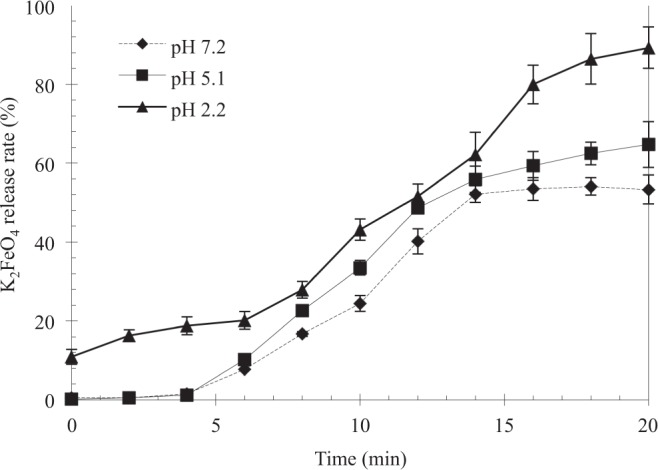
The degree of Fe^VI^ released from the chitosan capsule under different pH values.

**Figure 6:**
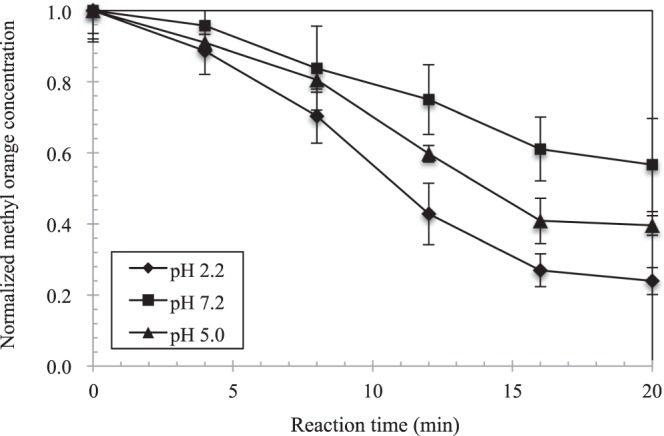
Removal efficiency of methyl orange using encapsulated ferrate (Experimental conditions: methyl orange concentration = 5 mg/l, encapsulated ferrate sample = 1 g, solution volume = 100 ml).

To study the influence of pH on the reaction of encapsulated Fe^VI^ with methyl orange, a fixed dosage of the encapsulated Fe^VI^ was mixed with methyl orange solution (5 mg/l) at pH values of 2.2, 5.0 and 7.2 and continuously monitored over a 20-min period. As shown in Fig. 5 using chitosan/CO/Fe^VI^ system for illustration, the degree of Fe^VI^ released from the pellets increased markedly with decreases in the pH value. We observed about 50% Fe^VI^ released after 20 min at a pH of 7.2, and more than 60% and 80% releases for a pH value of 5.0 and 2.2, respectively. It is worth noting that, while these Fe^VI^-release temporal profiles generally followed the S-shape pattern discussed earlier, the initial lag stage (Stage I) at pH 2.2 was not as pronounced as they were for the pH values of 5.0 and 7.2. In fact, chitosan swelling appeared to have occurred immediately after exposing to the solution at pH 2.2 and might have also allowed an accelerated diffusion of Fe^VI^ particles through the buffer layer. The behavior of the pH-controlled release was also reflected in the methyl orange removal patterns shown in Fig. 6, corresponding to the identical experimental conditions as in Fig. 5. The experimental results show a faster rate and a higher degree of removal of methyl orange at a lower pH, with about 75% removal for pH 2.2 after 20 min of reaction as compared to 60% for pH 5.0 and only 40% for pH 7.2. There were no visible lag periods in the initial stage of the reaction as seen in the Fe^VI^ release, presumably because methyl orange adsorption on chitosan occurred as soon as the pellets were mixed with the methyl orange solution.

We also postulate that, as Fe^VI^ penetrates the buffer, the continuous oxidation of the adsorbed methyl orange molecules and the re-adsorption onto the vacant chitosan sites form a dynamic process. In a typical experimental run, a 100-ml solution containing 1 g of pellets covering an average of 20 mg of K_2_FeO_4_ in contact with 5 mg/l methyl orange would give a total of 0.5 mg methyl orange molecules available for reaction. Approximating a methyl orange removal at 60% after 20 min of reaction with the chitosan-encapsulated pellets (referring to Fig. 6), an amount of 0.3 mg would have been removed from the solution. Also, with an equilibrium adsorption capacity of methyl orange at about 0.2 mg/g (Fig S7), about 0.2 mg methyl orange would have been adsorbed onto chitosan at equilibrium. However, observing the time to reach equilibrium needing about 2 hours, it is likely that only a fraction of the equilibrium adsorption capacity was actually attained within the initial 20 min. We therefore deduce that adsorption alone cannot explain the mass removal of methyl orange from the solution, and that the released Fe^VI^ is responsible for the removed amount observed in the experiments. Given the concurrent effects of the adsorption and degradation dynamics of methyl orange, we were not able to calculate the mass distribution of Fe^VI^ consumed by the oxidations of surface-bound methyl orange and bare chitosan surface, as well as those potentially released into the solution.

### Implications to environmental decontamination

As with most other oxidants, ferrate applications for the decontamination of environmental pollutants are limited to water and wastewater purification. In a recent review article, Rai and coworkers^52^ suggested that Fe^VI^-based materials also have the potential for soil and groundwater cleanup on the merit of its strong oxidative property to degrade recalcitrant soil contaminants, though the authors gave no further details concerning the method of delivery. Encapsulation of chemicals has been manufactured and tested to achieve the controlled release of active chemical for groundwater treatment. As reviewed by O’Connor and coworkers^53^ on the subject of *in-situ* groundwater remediation with controlled release materials, they indicate that technology applications both *in situ* bioremediation and *in-situ* chemical treatment have roughly tripled over the past decade than those in the decade prior. Depending on the functionality of the *in situ* treatment, the reactive materials can be oxidants, oxygen, substrate (to provide O_2_ or electron donors for growth stimulation of aerobic bacteria), or even microbial culture. There is little doubt that the potential application of controlled release of active chemicals for subsurface decontamination has gained growing technical popularity, though important factors such as the time-scale of treatment and the environmental conditions need to be considered.

A comparison of the controlled-release studies for environmental remediation is presented in Table 2. Most existing studies reported in the literature used a petroleum derivative as the sole or as a primary component of the shell formation. These shell formations typically last weeks or months when applied to remediate groundwater contamination. For remediating complex groundwater contaminations, especially those involved dense non-aqueous phase liquid (DNAPL) such as trichloroethylene (TCE), treatment time-scales are typically in the order of years, entailing longevity of the binding material of the *in situ* chemicals to withstand numerous attrition factors that gradually deteriorate the protective function of binders. While potassium permanganate (KMnO_4_) has demonstrated effective oxidation of recalcitrant organic contaminants, paraffin wax possessing the attributes of being unreactive and morphologically sturdy has been an increasingly popular choice of material to contain reactive reagents in the forms of candle^54^, pellet^55^, and microcapsule^56^. Other types of chemically resistant polymers such as polycaprolactone^57^ and polyurethane^58^, have also been applied as the primary binding material for similar reasons. These encapsulated particles or pellets gradually dissolved in solutions of designation and are invariably effective for single uses. Paraffin candles entrapping core oxidants, due to their larger volume, can be used multiple times, even though they are never “recharged” or “regenerated” with the replenishment of fresh oxidant.

**Table 2:** A comparison of the existing studies using various types of core and shell combination for controlled release applications.

Core material	Shell material	Encapsulation method	Duration of sustained release	Compound(s) challenged	Degradation rate	Ref.
K_2_FeO_4_	Paraffin	Molten/cooling	108 h	Trichloroethylene (TCE)	>90% after 60 min at pH 4.0–6.0; ~60% after 150 min at pH 10	^34^
K_2_FeO_4_	Ethyl cellulose/ paraffin	Phase separation	90% release after ~1.6 months at mass ratio (shell:core) of 1:1; complete release ~3 min in pure phase PCE	2-sec-butyl-4,6-dinitrophenol	~93% after 80 min at pH 6.5; ~70% at pH 4.0; ~35% at pH 10	^35^
KMnO_4_	Paraffin			Perchloroethylene (PCE)	KMnO_4_ rapidly released into pure phase PCE (∼3 min) as the paraffin wax completely dissolved. Encapsulated KMnO_4_ particles preferentially accumulated at the PCE-water interface.	^55^
KMnO_4_	Stearic acid	Oil phase separation	~30% release after 240 h using KMnO_4_-to-stearic acid mass ratio of 1:3; ~60% at mass ratio of 1:1	TCE	90% TCE (c_0_ = 10 mg/l) degraded at pH 2.9 in 2 h; ~75% at pH 6.8–8.8. Degradation lasts up to 12 h, using 17.5 mg pellets with KMnO_4_-to-stearic acid mass ratio of 1:3.	^56^
KMnO_4_	Polycaprolactone/ starch	Melt blending to form cubes	~64% after 76 d, mostly in the first 10 d	TCE	~95% removal (c0 = 0.5 mg/L) using column tests, effective up to ~100 pore volumes	^57^
Na_2_S_2_O_8_	Paraffin	Melt blending to form candles	~180 mg/d	Benzene BTEX	~80% after 6 h (c0 = 0.5 mM) using fresh candle; ~35% benzene (0.1 mM) ~50% toluene (0.08 mM) ~55% ethylbenzene (0.07 mM) ~60% xylene (0.07 mM)	^54^

For situations where remediation of the unsaturated zone of subsurface contamination (e.g., early leaching period of agrochemical and other emerging contaminants), the time-scale requirement (in days) for the remediation can be substantially shorter than remediation of DNAPL. There are also instances where the delayed release of a chemical (in minutes) is desired for wastewater treatment. In these situations, encapsulation of oxidants with petroleum-based polymers may not be suitable, as releasing oxidant from its binder may be very slow. Alternatively, naturally-occurring polysaccharides (e.g., cellulose, dextran, pectin, alginic acid, agar, agarose, chitosan, and carrageenan) can be used as the binder that is also more environmentally friendly than their petroleum counterparts. Particularly, chitosan derivatives are nontoxic, biocompatible, and biodegradable – A major reason they have been widely used in pharmaceutical and product to control the delivery rate of conventional drugs, protein drugs, and bioactive compounds. We have demonstrated in the present study that, by architecting the encapsulation of a powerful oxidant (in Fe^VI^) with a natural biopolymer (in chitosan) in combination with a long-chained nonpolar hydrocarbon buffer medium, it is possible to use an environmentally benign wall material while controlling the rate of release of the oxidant by the buffer medium. The time-scale of the controlled release can range from minutes (CO), hours (MO) to days (OA) depending on the type of hydrocarbon buffer medium of choice.

## Conclusions

We have successfully demonstrated the potential to use a natural material in chitosan as a wall material to form a shell layer to encapsulate Fe^VI^, separated by another hydrocarbon layer protecting chitosan to be directly attacked by its ferrate content.

Preliminary tests indicated that the chitosan-ferrate capsules remained stable for at least 20 days, with no significant loss of ferrate content when the particles were exposed in an open-air condition. The ferrate could be released in a controlled manner through adjusting the pH of a solution, as demonstrated by the control release study that indicated accelerated ferrate release when the microcapsules were placed in solutions with greater acidity. Results of degradation of methyl orange using the chitosan-ferrate microcapsules in solution with various pH values was consistent with the ferrate release study. The greater extent of degradation was observed for methyl orange when the microcapsules were placed in more acidic solutions.

## Methods

### Preparation of Fe^VI^/buffer/chitosan pellets

Commercial potassium ferrate (Tianjin Weiyi Chemical, China), which had a purity of about 12% experimentally determined following a method previously reported^59,60^. Encapsulation with chitosan was prepared by coacervation technique and the liquid hardening method. The procedural simplicity and consistency at laboratory-scale level render coacervation as the method of choice. The tested chemical parameters included chitosan (75–85% deacetylated, Sigma-Aldrich) as the wall material (concentrations 0.5%, 1%, 1.5% in 1% glacial acetic acid) and NaOH the hardening agent (concentrations 5%, 10%, 15%).

A carbohydrate layer was added between the chitosan wall material and ferrate via an ultrasound-assisted co-mixing step. The additional layer was designed to prevent unwanted oxidation, as ferrate that could attack the hydroxyl and amino functional groups in chitosan. Oleic acid (OA) (C_18_H_34_O_2_, 90%, Sigma-Aldrich), refined coconut oil (CO) (a mixture of C8-C16 saturated fatty acids, mainly lauric acid and myristic acid) and mineral oil (MO) (mixtures of C9 and higher alkanes) were the three types of hydrocarbon buffers experimented. To synthesise capsules using OA and MO as buffers, potassium ferrate was directly mixed with the buffering solution by sonification (Q700, Qsonica, USA). The mixture was added to the 1% chitosan solution and stirred thoroughly. The Fe^VI^/buffer/chitosan mixture was then syringe-injected into NaOH as the hardening agent using a gauge-18 hypodermic needle. Additionally, considering the chitosan solution is insoluble to either OA or MO, a non-ionic surfactant (Tween 80, Sigma-Aldrich) was added as an emulsifier to improve the homogeneity of the mixture. In this case, the surfactant (1 ml) was added simultaneously with the ferrate solution to the buffering solution. Upon the formation of the microcapsules, they were placed in a freeze dryer (FD-1, Eyela, Japan) for drying for 3 h.

The presence of fatty acids as the main components of CO makes it characteristic of high boiling point. When heating above room temperature (25 °C) CO is in liquid form, but it solidifies at a temperature below this. Consequently, capsules with a two-layer formation can be synthesised by first mixing potassium ferrate powders in CO in the liquid state, followed by solidifying the Fe^VI^/CO droplets using a syringe to form particles in a cooling bath. The droplets were then coated with chitosan by immersing in 1% chitosan solution, before finally forming pellets by syringe-injecting the Fe^VI^/CO/chitosan into the hardening solution.

The prepared pellets were characterised by scanning electron microscopy (SEM), X-ray diffraction (XRD), and Fourier Transform Infrared Spectroscopy (FTIR). Detail information on these instrumental analyses is included in Supplementary Material S1.

### Ferrate capsules stability and controlled release studies

The stability of Fe^VI^ encapsulated in the as-prepared pellets was performed by exposing the pellets in ambient air for at least 20 days. Dry pellets sample of 0.1 g was randomly taken every 48 h, ground and placed in 100 ml of deionised water (25 °C, pH 7.0), and the concentration of Fe^VI^ was quantified by measuring the diffusion reflectance at the wavelength of 510 nm^61^ using a UV-Vis spectrophotometer (V-630, Jasco, Japan).

The stability of the Fe^VI^ entrapped in the buffering agents was tested as follows. Multiple samples containing an identical amount of potassium ferrate (50 mg) covered with a buffering agent (5 ml) were prepared in 20 ml vials. At each 20 min interval, one sample was taken and mixed with 15 ml of DI water. The mixture was then filtered with a 0.22 µm disc membrane, and the residual Fe^VI^ was quantitated using the UV-Vis spectrophotometric method depicted earlier. Furthermore, for the solid Fe^VI^/CO droplets (3 g in 10 ml), a fixed amount of samples (75 mg) were taken in a 20 min interval and placed in vials containing 10 ml of water and heated to 40 °C to melt the droplets. The residual Fe^VI^ released into the solution was then measured by the same method. Each test was performed in duplicate, and the average value and its range were presented in Fig. 1.

The controlled releases studies were conducted by weighing 0.1 g pellet sample into separate flasks containing deionised water whose pH was adjusted by sulfuric acid to 7.0, 5.2, and 2.1, respectively. The experiments were performed at room temperature (20–25 °C) under a mild stirring condition. Samples were taken every 2 min in the initial 20 min of the release test, and 10 min afterward, to observe the release rate of Fe^VI^ analyzed spectrophotometrically as described previously. Each experimental run were performed in duplicate.

### Degradation by chitosan-encapsulated Fe^VI^ pellets

The oxidative reaction between encapsulated Fe^VI^ samples and methyl orange was carried out using 250 mL glass reactors in which methyl orange solution was agitated with magnetic stirrers when an accurate weight of the capsules was added. The reactors were mechanically stirred for selected periods. For analysis, the samples were stirred for 20 min before analysis. All the experiments were carried out at room temperature (25 ± 1 °C). The concentration of methyl orange was determined by UV-Vis absorption spectrometer (Jasco V-63, Japan); the absorption spectrum shows an absorption peak at 470 nm^62^. Methyl orange adsorption by chitosan was studied separately from the oxidation experiments by Fe^VI^ to elucidate the amount of the methyl orange oxidatively removed. All experimental runs were performed in duplicate, unless there were any data points in a particular run that exceeded 10% of the average value. In such cases, a third run was conducted to verify the accuracy of the experiments. Detail information can be seen in Supplementary Material S3 and S4. Oxidative reaction time, encapsulated ferrate were investigated for their effect on the oxidation performance regarding methyl orange removal as a function of reaction time.

## Supplementary information

Supplementary Information

## Data Availability

All data generated or analysed during this study are included in this published article (and its Supplementary Information files).
