# Supplementary material for: Chitosan Encapsulation of FerrateVI for Controlled Release to Water:Mechanistic Insights and Degradation of Organic Contaminant
Source: Sci Rep. 2019 Dec 4;9:18268. doi: 10.1038/s41598-019-54798-4 (PMC6892851; doi:10.1038/s41598-019-54798-4)
Supplement: Supplementary file 1 — Supplementary Information [file 41598_2019_54798_MOESM1_ESM.docx]

**Supplementary Materials**

for

**Article Title:**

Chitosan Encapsulation of Ferrate^VI^ for Controlled Release to Water:

Mechanistic Insights and Degradation of Organic Contaminant

Bo-Yen Chen^1^, Hsuen-Wen Kuo^1^, Virender K. Sharma^2^, Walter Den^3^*

1. Department of Environmental Science and Engineering, Tunghai University, Taichung, Taiwan R.O.C. ([jnes50312@gmail.com](mailto:jnes50312@gmail.com))

2. Department of Environmental and Occupational Health, School of Public Health,

Texas A&M University, College Station, Texas, U.S.A. ([vsharma@sph.tamhsc.edu](mailto:vsharma@sph.tamhsc.edu))

3. Institute for Water Resources Science and Technology, Department of Science and Mathematics, Texas A&M University-San Antonio, San Antonio, Texas, U.S.A. ([wden@tamusa.edu](mailto:wden@tamusa.edu))

**S1. Materials characterizations**

X-ray diffraction (XRD) was used to determine the phase and structure of the obtained products of Fe^VI^-encapsulated pellets. The XRD patterns were obtained using CuKα radiation on Philips X’pert Pro (Model Philips X’PERT MPD, USA) diffractometer. The X-ray was generated with a current of 40 mA and a potential of 40 kV. The samples were scanned from 20 to 80 degrees (2θ) in steps of 0.02 degrees per second. Verification of the capsule contents was aided by employing Fourier Transform Infrared Spectroscopy (FTIR) to characterize the functional groups distinctive to the binding material (i.e., chitosan), the protective layer and the active ingredient (i.e., Fe^VI^).

The morphologies of samples were observed with field emission scanning electron microscopy (FE-SEM) (S-3400N, Hitachi, Japan) operated in a high-vacuum mode with secondary electron image conditions and the electron micrograph technique. The SEM operated at 3 kV to 30 kV with an ultimate resolution of 10 nm to 3.0 nm. The magnification range from 5 to 300,000 times the original size.

| (a)  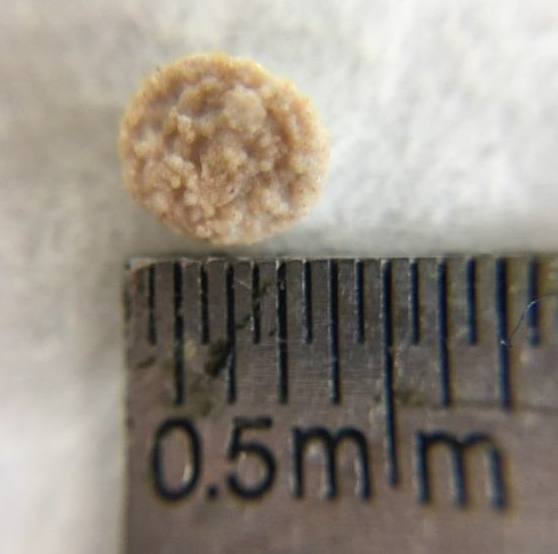 | 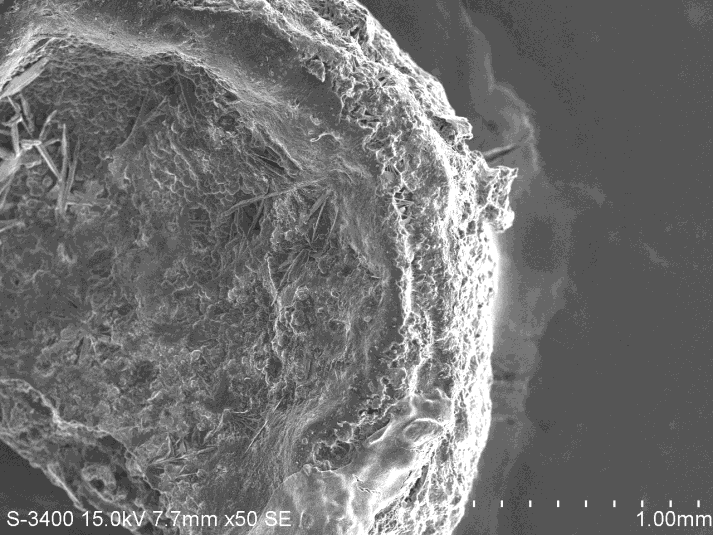 |
| --- | --- |
| (b)  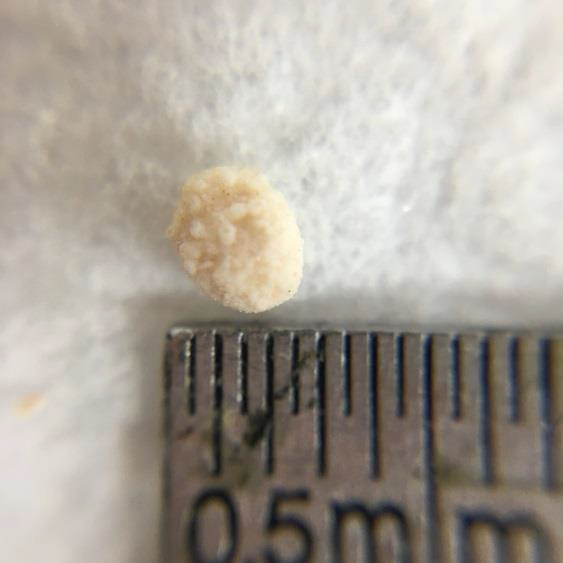 | 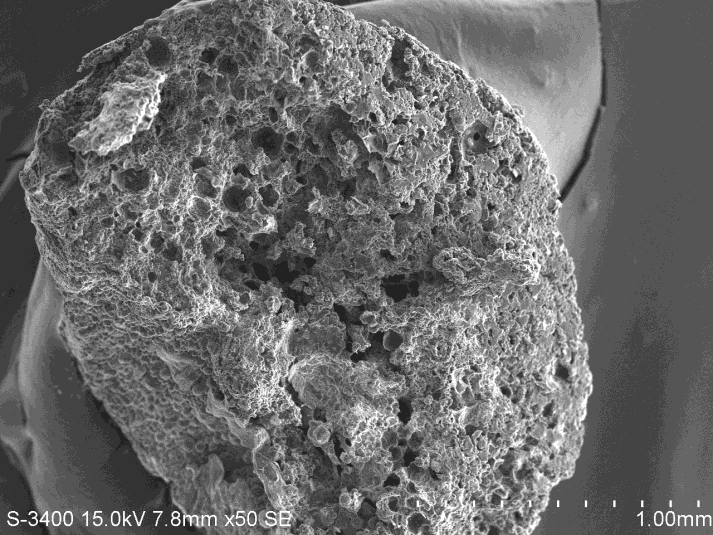 |
| (c)  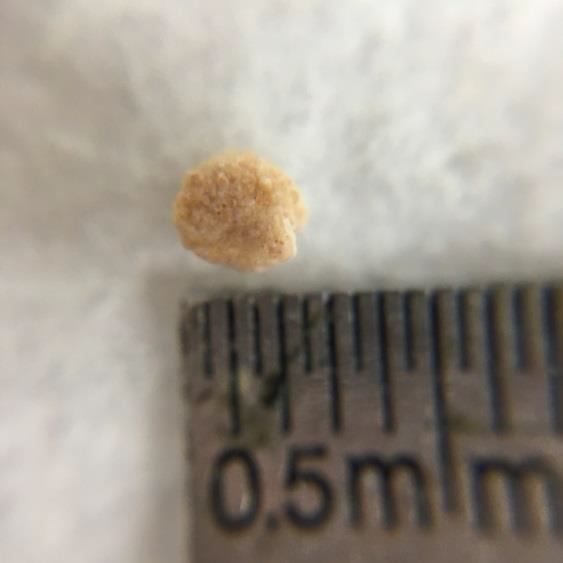 |  |
| (d)  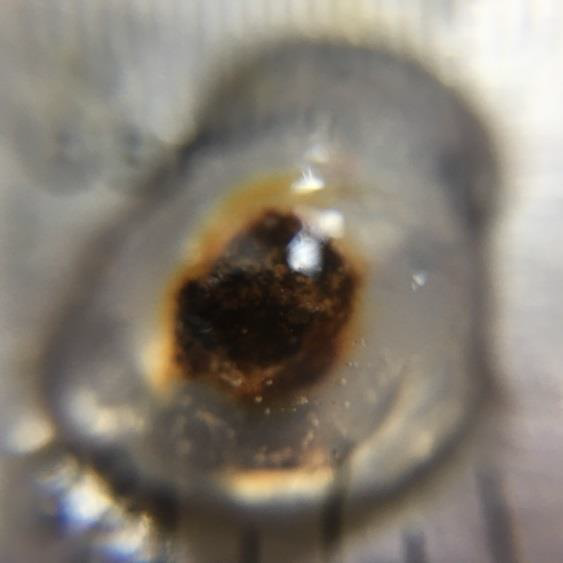 | (e)  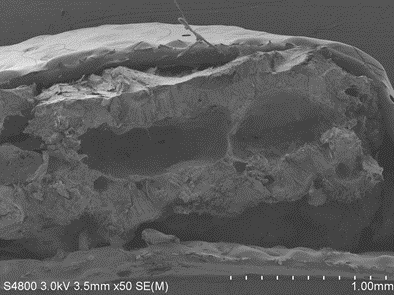 |

Figure S1. Photo and SEM images (50x magnification) for (a) Fe^VI^/OA/chitosan; (b) Fe^VI^/OA (with Tween 80)/chitosan; (c) Fe^VI^/CO/chitosan; and (d) Fe^VI^/CO droplets; (e) cross-section view showing chitosan as the outer wall, OA as the filler, and the hollow core where K_2_FeO_4_ occupied.


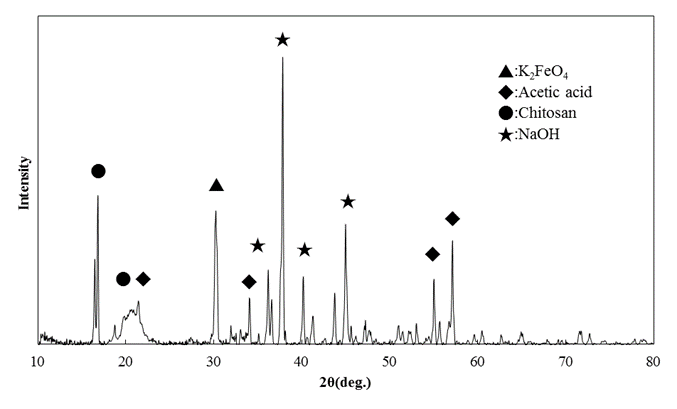


Figure S2. Representative X-ray diffractometry of a chitosan-encapsulated Fe^VI^ pellet. Characteristics peaks of both potassium ferrate^VI^ and chitosan are clearly present.


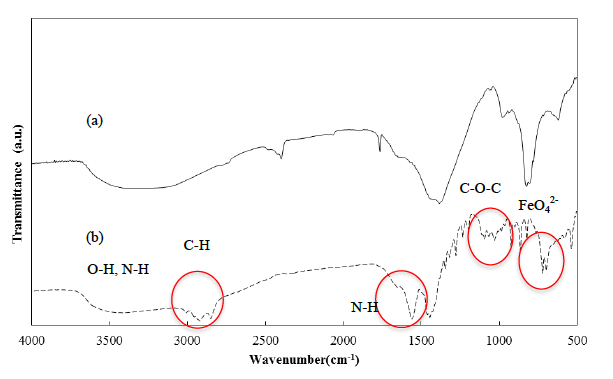


Figure S3. A typical FTIR spectra of a chitosan-encapsulated Fe^VI^ pellet: (a) K_2_FeO_4_ powder; and (b) chitosan-encapsulated K_2_FeO_4_.

**S2. Stability studies**

S4. The amount of chitosan needed to reduce Fe^VI^ to Fe^III^ during the titration experiment of K_2_FeO_4_ solutions (80-400 mg/l) with 0.5% chitosan (w/v in acetic acid). The linear function suggests that a stoichiometric reaction between chitosan and K_2_FeO_4_.


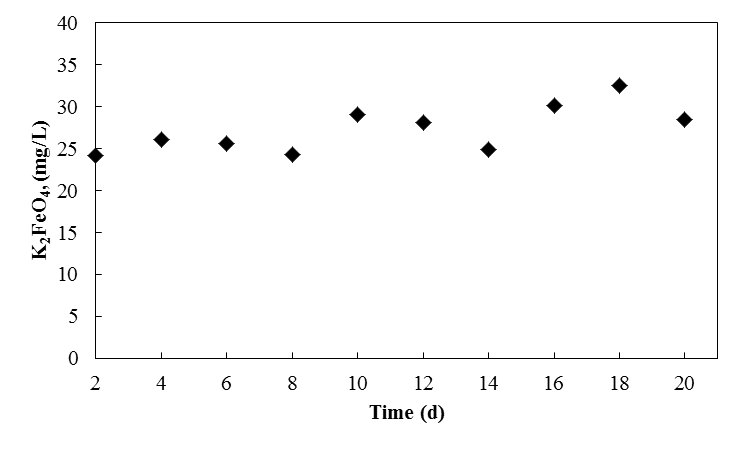


Figure S5. Remaining K_2_FeO_4_ concentration in the encapsulated ferrate over the first 20 days after exposing to ambient air.

**S3. Adsorption of methyl orange by chitosan**

40 mg of chitosan powders were mixed with 20 ml of methyl orange at concentrations between 5 mg/l and 30 mg/l. The solutions were adjusted to various pH levels (2, 5, 6.5, 10) using 0.1M NaOH or 0.1M HCl. A sample was taken every 2 h, and solids were removed by syringe filtration (0.45 µm), whereas the methyl orange concentration in the filtrate was determined spectrophotometrically at 465 nm. The adsorption experiments continued until reaching an equilibrium state between the sorbent and sorbate.


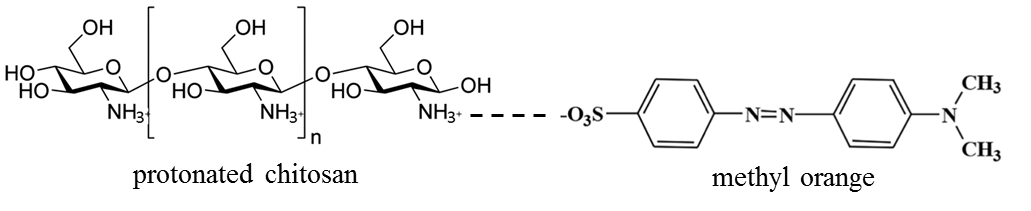


Figure S6. Possible mechanism of adsorption by ionic bonding between chitosan and

methyl orange molecules.


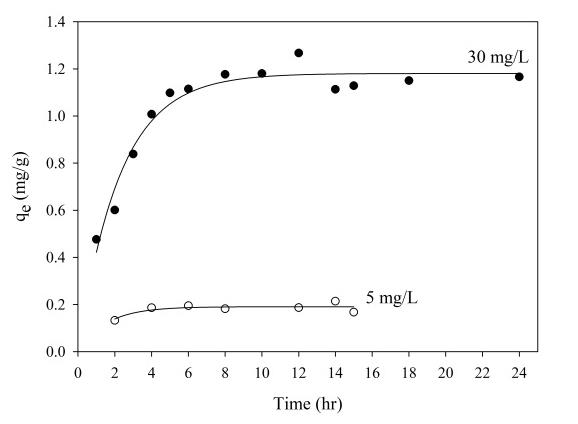


Figure S7. Adsorption equilibrium profiles of methyl orange at low (5 mg/l) and high

(30 mg/l) by chitosan at pH 6.5.

**S4. *Oxidation of methyl orange by Fe^VI^***

The oxidative power of Fe^VI^ to methyl orange was determined by directly mixing the various amount of potassium ferrate powders with 5 mg/l methyl orange solution, forming Fe^VI^ concentrations between 6 mg/l and 36 mg/l. The initial sample was taken immediately after mixing, and every 2 min thereafter. Sample treatment for determining methyl orange concentration followed the procedure mentioned above.


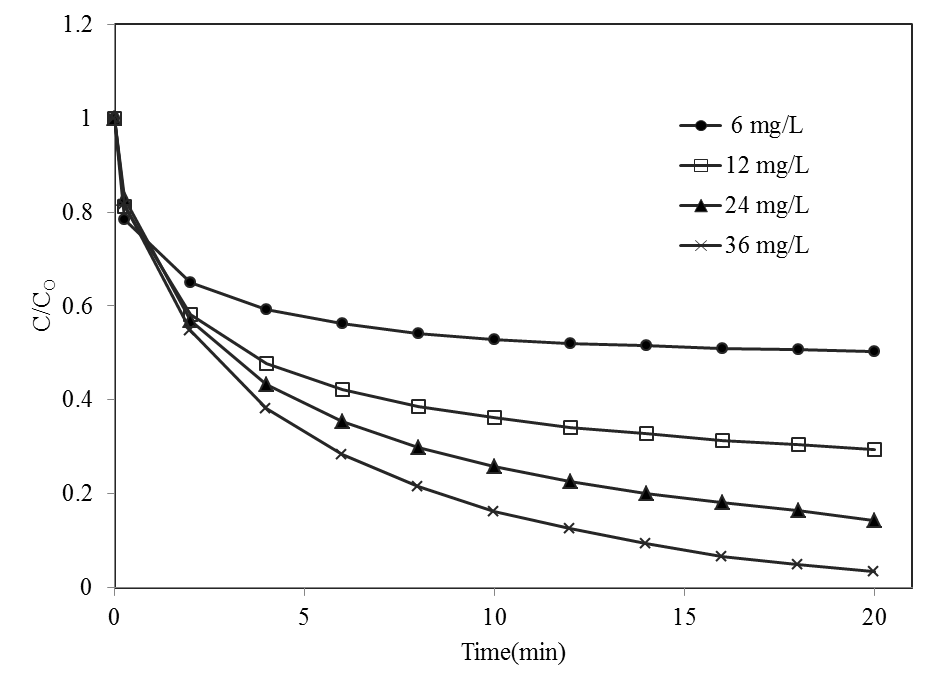


Figure S8. Methyl orange oxidation profiles as a function of reaction time under various concentration of Fe^VI^ without buffering and encapsulation.

**S5. Removal of methyl orange with buffered Fe^VI^**

Figure S9. The removal rate of methyl orange in the initial 20 min of reaction with K_2_FeO_4_ buffered with OA, CO, and MO (top to bottom, K_2_FeO_4_ dosage at 6 mg/l, 12 mg/l, 24 mg/l, and 36 mg/l.)
